# Exploring the link between obesity and hypothyroidism in autoimmune thyroid diseases: a metabolic perspective

**DOI:** 10.3389/fmolb.2024.1379124

**Published:** 2024-04-22

**Authors:** Mengzhe Jing, Shanshan Shao, Shizhan Ma, Ling Gao, Qian Wang, Meng Zhou

**Affiliations:** ^1^ Key Laboratory of Endocrine Glucose & Lipids Metabolism and Brain Aging, Ministry of Education, Department of Endocrinology, Shandong Provincial Hospital Affiliated to Shandong First Medical University, Jinan, Shandong, China; ^2^ Shandong Key Laboratory of Endocrinology and Lipid Metabolism, Jinan, Shandong, China; ^3^ Shandong Institute of Endocrine and Metabolic Disease, Jinan, Shandong, China; ^4^ Department of Ultrasound, Shandong Provincial Hospital Affiliated to Shandong First Medical University, Jinan, Shandong, China

**Keywords:** metabolic obesity phenotypes, hypothyroidism, triglyceride, metabolism, autoimmune thyroid disease

## Abstract

**Background:** The management of primary hypothyroidism demands a comprehensive approach that encompasses both the implications of autoimmune thyroid disease and the distinct effects posed by obesity and metabolic irregularities. Despite its clinical importance, the interplay between obesity and hypothyroidism, especially in the context of metabolic perspectives, is insufficiently explored in existing research. This study endeavors to classify hypothyroidism by considering the presence of autoimmune thyroid disease and to examine its correlation with various metabolic obesity phenotypes.

**Method:** This research was conducted by analyzing data from 1,170 individuals enrolled in the Thyroid Disease Database of Shandong Provincial Hospital. We assessed four distinct metabolic health statuses among the participants: Metabolically Healthy No Obese Metabolically Healthy Obese Metabolically Unhealthy No Obese and Metabolically Unhealthy Obese Utilizing logistic regression, we investigated the association between various metabolic obesity phenotypes and hypothyroidism.

**Results:** The study revealed a significant correlation between the Metabolically Unhealthy Obese (MUO) phenotype and hypothyroidism, particularly among women who do not have thyroid autoimmunity. Notably, the Metabolically Unhealthy No Obese (MUNO) phenotype showed a significant association with hypothyroidism in individuals with thyroid autoimmunity, with a pronounced prevalence in women. Furthermore, elevated levels of triglycerides and blood glucose were found to be significantly associated with hypothyroidism in men with thyroid autoimmunity and in women without thyroid autoimmunity.

**Conclusion:** Effective treatment of hypothyroidism requires a thorough understanding of the process of thyroid autoimmune development. In patients without concurrent thyroid autoimmunity, there is a notable correlation between obesity and metabolic issues with reduced thyroid function. Conversely, for patients with thyroid autoimmunity, a focused approach on managing metabolic abnormalities, especially triglyceride levels, is crucial.

## 1 Introduction

Hypothyroidism, prevalent worldwide, shows distinct geographical prevalence disparities, ranging from 4.6% to 9.5% (Canaris et al., 2000; Hollowell et al., 2002; Garmendia Madariaga et al., 2014). The prolonged impact of hypothyroidism can lead to severe health issues due to the critical role of thyroid hormones in regulating multiple physiological processes and ensuring bodily equilibrium. Such complications encompass an elevated risk of cardiovascular diseases, metabolic dysfunctions, neuropsychiatric conditions, and reproductive health challenges (Dugbartey, 1998; Biondi and Klein, 2004; Stabouli et al., 2010a; Stabouli et al., 2010b; Rodondi et al., 2010; Su et al., 2022). A key driver behind primary hypothyroidism is thyroid autoimmunity, which stands in stark contrast to non-autoimmune hypothyroidism in terms of causation, clinical characteristics, and treatment efficacy (Guerri et al., 2019). This underscores the necessity for research that stratifies hypothyroidism based on the presence or absence of thyroid autoimmunity, aiming for a deeper insight into its holistic prevention and treatment strategies, thus highlighting its clinical significance.

In parallel with swift economic development and lifestyle transformations, obesity has become increasingly prevalent globally (Ng et al., 2014; Afshin et al., 2017). The rise in obesity rates is linked to a greater risk of developing diseases such as diabetes, cardiovascular conditions, and cancer, as corroborated by several studies (Kahn et al., 2006; Van Gaal et al., 2006; Khandekar et al., 2011). Furthermore, recent research highlights obesity’s critical role in the evolution and aggravation of hypothyroidism (Santini et al., 2014; Lee et al., 2015; Licenziati et al., 2019). Evidence from a synthesis of 22 studies indicates a heightened hypothyroidism risk in obese individuals, encompassing both overt and subclinical forms (Song et al., 2019). It is noteworthy that different obesity phenotypes are associated with varying levels of disease risk, often accompanied by metabolic imbalances (Geetha et al., 2011; van Vliet-Ostaptchouk et al., 2014; Jung et al., 2017). Our previous investigations explored the relationship between hypothyroidism and gastrointestinal polyps across varied metabolic obesity categories. These findings advocate for a nuanced approach in obesity classification based on metabolic parameters, offering a novel perspective for patient categorization and management (Wang et al., 2021; Cheng et al., 2022).

In this study, participants were meticulously classified based on their distinct metabolic abnormalities, while hypothyroidism categorization was guided by the prevalence of autoimmune thyroid disorders. Such systematic stratification was instrumental in facilitating an initial investigation into the nexus between various metabolic obesity phenotypes and hypothyroidism. Moreover, the research extended to probe the relevance and potential influence of metabolic factors on the development and progression of hypothyroidism. This methodological approach was designed to foster a 70 comprehensive understanding of the complex interplay between metabolic health and thyroid 71 function, with a particular emphasis on autoimmune thyroid disorders.

## 2 Materials and methods

### 2.1 Study population

**FIGURE 1:**
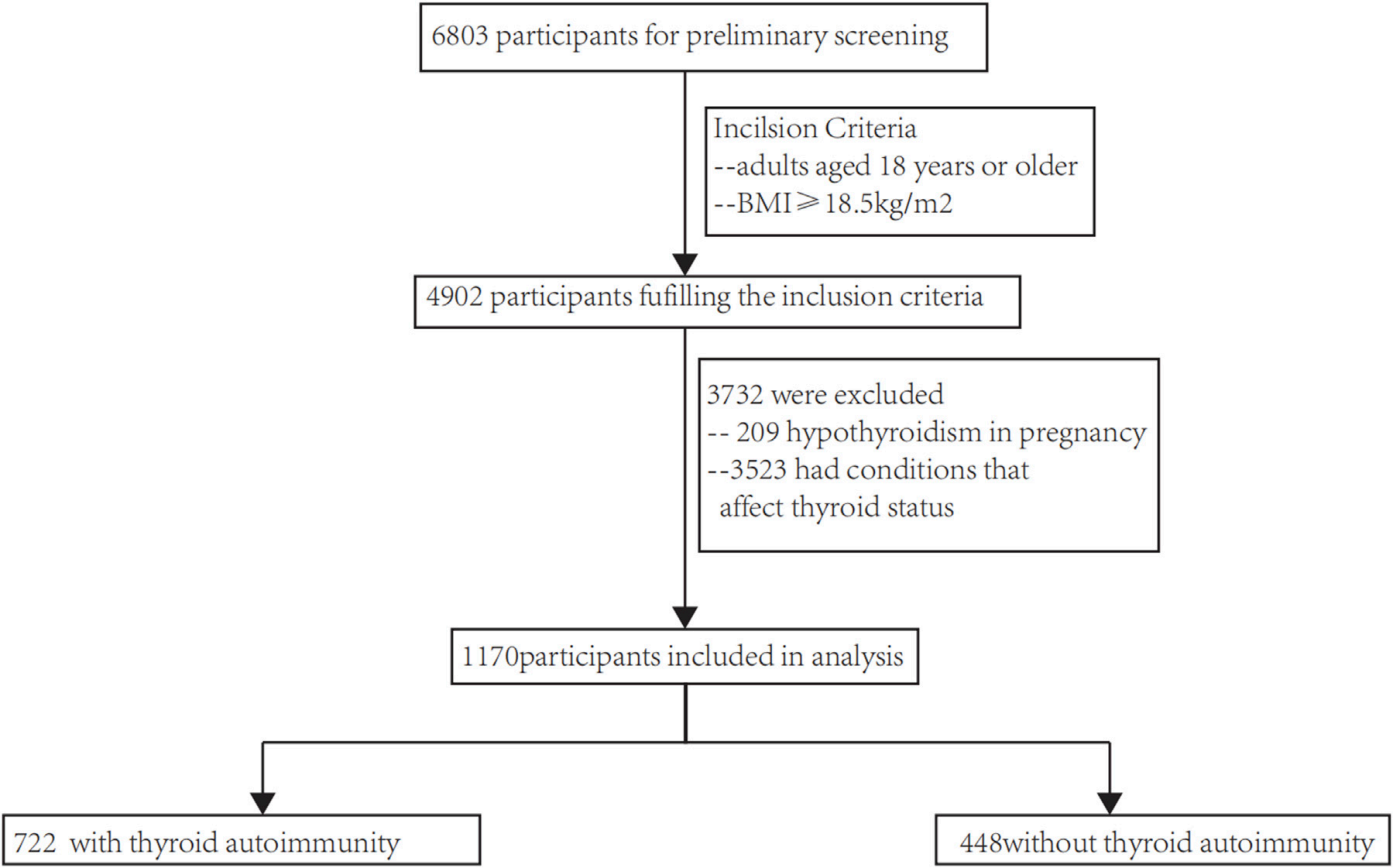
The fiow chart of enrollment.

A database of thyroid disease patients at Shandong Provincial Hospital was used to select participants. The inclusion criteria consisted of 1) individuals who were at least 18 years old, and 2) a Body Mass Index (BMI) above 18.5 kg/m^2^. The exclusion criteria consisted of 1) absence of blood lipids (such as triglycerides (TG) and high-density lipoprotein cholesterol (HDL-C)), blood pressure (Diastolic Blood Pressure (DBP) and Systolic Blood Pressure (SBP)) and fasting blood glucose (FPG); and 2) the presence of conditions that influence thyroid status, such as the utilization of medications that impact thyroid function, treatment with radioactive iodine, thyroid removal surgery, severe liver or kidney dysfunction, malignant tumors, *etc.* In the end, a total of 1,170 patients fulfilled our requirements (as depicted in Figure 1), and every single one (100%) was incorporated into the examination. Biomedical Ethics Committee approval, compliance with the 1975 Helsinki Declaration, and authorization by Shandong Provincial Hospital’s Human Research Committee were obtained for this study. According to STROBE (Strengthening the Reporting of Observational Studies in Epidemiology), this article presents the results of an observational investigation.

### 2.2 Data acquisition

(1) General Data Extraction: Demographic information and medical history (including hypertension, coronary heart disease, diabetes, thyroid family history, *etc.*) of target patients were extracted from our hospital’s thyroid-specific disease database. Gender was categorized into female and male; age was divided into <45 years and ≥45 years.
(2) Height and weight data were extracted to calculate BMI.
(3) Blood biochemical data were extracted: FPG, TG, HDL-C, *etc.*

For thyroid function, thyroid-related hormones (such as Free Triiodothyronine (FT3), Free Thyroxine (FT4), Thyroid Stimulating Hormone (TSH)) and thyroid autoantibodies (such as Thyroid Peroxidase Antibody (TPO-Ab), and Thyroglobulin Antibody (TgAb)) were measured.

To determine the occurrence of high blood pressure, data on DBP and SBP were extracted (Huang, 2009).

### 2.3 Definitions

Obesity is defined as a BMI greater than 28 kg/m^2^, which has been deemed to be a more appropriate criterion for determining obesity in Asian communities (Van Gaal et al., 2006). Metabolic unhealthiness is defined by the Diabetes Society of the Chinese Medical Association as meeting at least two out of the following four components (Ren et al., 2022): 1) A fasting plasma glucose level of at least 6.1 mmol/L; 2) a systolic blood pressure of at least 130 mm Hg or a diastolic blood pressure of at least 85 mm Hg; (3)The TG levels of males and females should not exceed 1.7 mmol per liter; 4) the high density lipoprotein cholesterol levels for males and females should not exceed 0.9 mmol per liter. Taking into account both the participants’ BMI and metabolic status, they were classified into four different metabolic obesity phenotypes: Metabolically Healthy No Obese (MHNO), defined as having a BMI of 18.5–28 kg/m^2^; with less than two metabolic disorders Metabolically Healthy; Metabolically Unhealthy No Obese (MUNO), defined as an individual with a BMI of 18.5–28 kg/m^2^ with more than 2 metabolic abnormalities; Metabolically Healthy Obese (MHO), defined as having a BMI of kg/m^2^ or higher with less than 2 metabolic abnormalities; and Metabolically Unhealthy Obese (MUO), defined as those with a BMI equal to or exceeding 28.0 kg/m^2^, accompanied by two or more metabolic abnormalities (Chaker et al., 2017). Young participants were defined as those <45 years, and older participants as ≥45 years.

Reference ranges for TSH were 0.27–4.2 mIU/L, FT4 was 12–22 pmol/L, FT3 was 3.1–6.8 pmol/L, and TPO-Ab was 0–60 IU/L. Normal thyroid function was defined as levels of TSH, FT4, and FT3 within the reference range. Hypothyroidism was characterized by TSH levels exceeding 4.2 mIU/L and FT4 levels below 12 pmol/L, indicating overt hypothyroidism. As another alternative, in cases where TSH levels exceeded 4.2 mIU/L without FT4 levels exceeding the reference range, hypothyroidism was also detected as subclinical (Huang, 2009).

### 2.4 Gender analysis

This study sought to explore the association between metabolic obesity phenotypes across genders and rates of hypothyroidism, given that hypothyroidism prevalence typically varies by gender.

### 2.5 Statistical analysis

In this study, mean x standard deviation, median x interquartile range (IQR), and proportions were used to summarize the basic characteristics of various metabolic obesity phenotypes. Analyzing continuous variables with normal and non-normal distributions by univariate analysis of variance (ANOVA) and Kruskal–Wallis tests was used for comparisons between groups. The significance values for multiple tests were adjusted using the Bonferroni correction. Categorical variables were compared using the chi-square test. Various metabolic obesity phenotypes were assessed using multivariate logistic regression models to determine the prevalence of hypothyroidism, with odds ratios (ORs) and 95% confidence intervals (CIs) being evaluated. Age, gender, alanine aminotransferase, aspartate aminotransferase, globulin, albumin, total protein, and lipoprotein were adjusted in the models. In order to assess potential confounding effects of gender, regression models were generated for comparing metabolic obesity phenotypes among different genders. We used SPSS version 26.0 to conduct the analyses. A statistically significant result was defined as a *p*-value less than 0.05.

## 3 Result

### 3.1 Flow chart

The flow chart of this study are performed in Figure 1. The research involved a grand total of 1,170 participants, consisting of 448 individuals who without autoimmune thyroid disease and 722 who suffer from autoimmune thyroid disease. There were 143 males and 305 females in the non-autoimmune group, while there were 173 males and 549 females in the autoimmune group. Both males and females were concentrated in the MHNO group, based on obesity phenotypes.

### 3.2 Clinical features

We collected and analyzed the clinical characteristics of all samples. In comparison to the MHNO and MHO phenotypes, individuals with the MUNO and MUO phenotypes exhibited markedly elevated levels of TG, SBP, DBP and FPG (all *p* < 0.01). Conversely, HDL-C was notably lower in the MUNO and MUO groups compared to the MHNO group (all *p* < 0.01). Table 1 shows that the MHO, MUNO, and MUO groups had elevated BMI, alanine aminotransferase (ALT), and total protein (TP) compared with the MHNO group (all *p* < 0.001) (Table 1).

**TABLE 1:** The baseline characteristics of the thyroid cancer study population.

Variables	MHNO	MUNO	MHO	MUO	*p*-value
No. of cases	526	299	151	194	
Age (years), mean (SD)	47.94 (15.85)	55.23 (13.98)	42.92 (16.07)	48.26 (14.67)	<0.001
BMI (kg/m^2^), mean (SD)	23.71 (2.46)	24.67 (2.17)	30.52 (2.38)	31.21 (3.05)	<0.001
Female, (n%)	399 (75.90)	207 (69.20)	199 (78.80)	129 (66.50)	<0.001
participants without thyroid autoimmunity, (n%)	209 (46.7)	91 (20.3)	71 (15.8)	77 (17.2)	0.004
participants with thyroid autoimmunity, (n%)	317 (43.9)	208 (28.8)	80 (11.1)	117 (16.2)	0.004
TSH(mIU/L), mean (SD)	8.11 (17.24)	7.77 (16.56)	9.14 (19.78)	10.53 (20.79)	0.376
T3 (pmol/L), mean (SD)	4.17 (0.90)	4.23 (0.80)	4.25 (0.98)	4.18 (0.93)	0.723
T4 (pmol/L), mean (SD)	14.10 (3.46)	14.40 (3.37)	14.61 (4.00)	13.83 (3.93)	0.173
TG (mmol/L), mean (SD)	1.22 (0.74)	2.16 (1.36)	1.56 (0.94)	2.77 (2.59)	<0.001
HDL-C (mmol/L), mean (SD)	1.49 (0.38)	1.25 (0.40)	1.49 (0.38)	1.16 (0.34)	<0.001
DBP (mmHg), mean (SD)	77.50 (11.13)	83.26 (10.90)	79.91 (10.76)	87.70 (11.67)	<0.001
SBP (mmHg), mean (SD)	123.86 (18.68)	138.60 (17.40)	125.53 (17.11)	141.18 (18.82)	<0.001
FPG (mmol/L), mean (SD)	5.46 (2.09)	8.20 (3.47)	5.20 (1.28)	8.07 (3.18)	<0.001
ALT (g/L), mean (SD)	20.32 (26.15)	23.25 (23.53)	25.08 (32.59)	29.05 (22.73)	0.001
AST (g/L), mean (SD)	23.56 (18.02)	24.81 (19.32)	27.02 (26.41)	27.85 (21.85)	0.055
ALB (g/L), mean (SD)	34.22 (19.61)	33.11 (19.57)	32.85 (19.45)	32.35 (19.45)	0.66
GLO (g/L), mean (SD)	28.99 (4.27)	29.50 (4.20)	29.01 (3.79)	29.49 (4.23)	0.281
LP (g/L), mean (SD)	70.05 (6.57)	71.09 (6.82)	69.66 (6.88)	71.93 (6.48)	0.002
Lpa (g/L), mean (SD)	0.4421 (4.32)	2.3465 (22.07)	1.56 (15.66)	3.83 (16.67)	0.101

### 3.3 Correlation analysis

#### 3.3.1 Correlation analysis between obesity metabolic phenotypes and hypothyroidism in whole individuals

**FIGURE 2:**
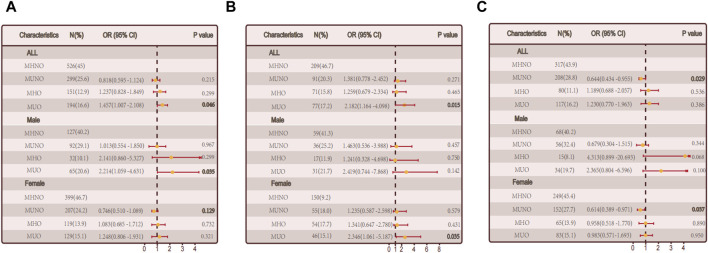
The relationship between metabolic obesity phenotypes and hypothyroidism. **(A)** Relationship between metabolic obesity phenotype and hypothyroidism in the general population. **(B)** Relationship between metabolic obesity phenotype and hypothyroidism in people without thyroid autoimmunity. **(C)** Relationship between metabolic obesity phenotype and hypothyroidism in thyroid autoimmune population.

Based on obesity metabolic phenotypes, the participants were divided into four distinct groups. MUO phenotype was significantly correlated with hypothyroidism in the overall population (*p* = 0.046) according to adjusted logistic regression models. In males (*p* = 0.035), a gender-segregated analysis revealed a significant association, whereas in females (*p* = 0.321) no such correlation was observed (Figure 2A).

#### 3.3.2 Correlation analysis between obesity metabolic phenotypes and hypothyroidism in non-autoimmune group and autoimmune group

We conducted a sub-group (non-autoimmune group and autoimmune groups) for the reason that autoimmune thyroiditis may have a effect. The MUO phenotype was positively correlated with hypothyroidism in the non-autoimmune group (*p* = 0.015). A notable connection was observed among females (*p* = 0.035) when considering gender stratification, while no significant correlation was found among males (*p* = 0.142). The link between obesity and non-autoimmune hypothyroidism may be mediated by metabolic abnormalities, as illustrated in Figure 2B. A significant correlation was found between the MHO phenotype and hypothyroidism in the autoimmune group (*p* = 0.029), while no significant correlations were observed in the other groups, indicating a possible involvement of metabolic factors in autoimmune hypothyroidism (Figure 2C).

### 3.4 Differential analysis in non-autoimmune group and autoimmune group

Additional examination was performed on the correlation between the quantity of metabolic irregularities and hypothyroidism. During our comprehensive analysis, we investigated the relationship between the quantity of metabolic irregularities and the prevalence of hypothyroidism.

**FIGURE 3:**
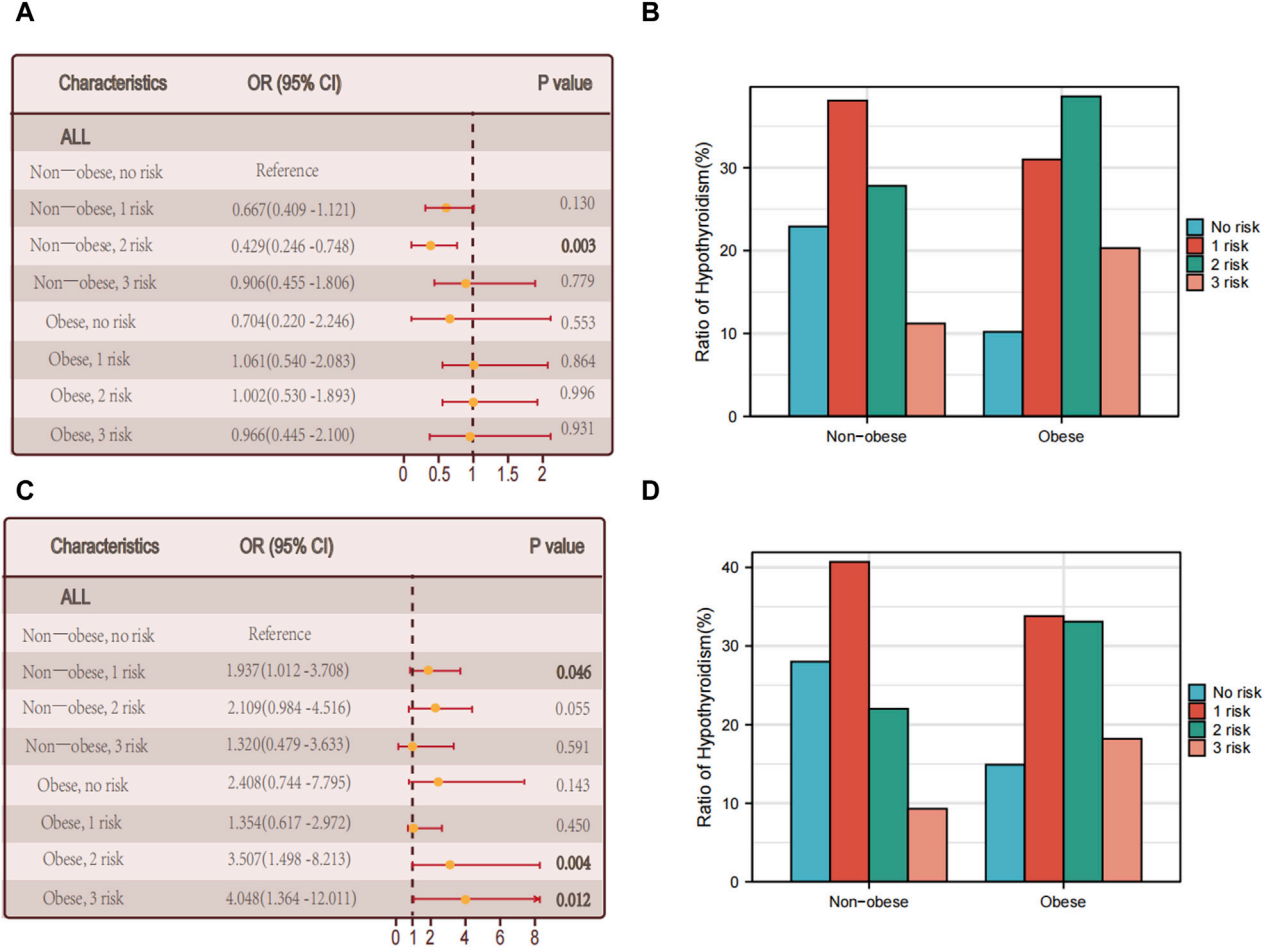
Relationship between hypothyroidism and the number of metabolic abnormalities. **(A)** The association between the number of metabolic abnormalities and hypothyroidism in all individuals in non-obese and obese populations with thyroid autoimmune diseases. **(B)** Proportion of multiple foci in the non-obese *versus* obese group of people with autoimmune thyroid diseases. **(C)** The association between the number of metabolic abnormalities and hypothyroidism in all individuals in non-obese and obese populations without thyroid autoimmune disease. **(D)** Proportion of non-obese *versus* obese multifoci in people without thyroid autoimmune disease.

The discovery is consistent with our prior analysis of the metabolic phenotype of obesity, indicating that intricate metabolic disorders might contribute to the interaction between obesity and hypothyroidism. Nevertheless, among individuals with autoimmune thyroid disorder, there was no notable association observed between the rise in metabolic irregularities and hypothyroidism (*p* = 0.931) (Figures 3A, B).

Among the obese individuals who did not have autoimmune thyroid disease, there was a notable correlation between the quantity of metabolic irregularities and hypothyroidism (*p* = 0.012) (see Figures 3C, D).

**FIGURE 4:**
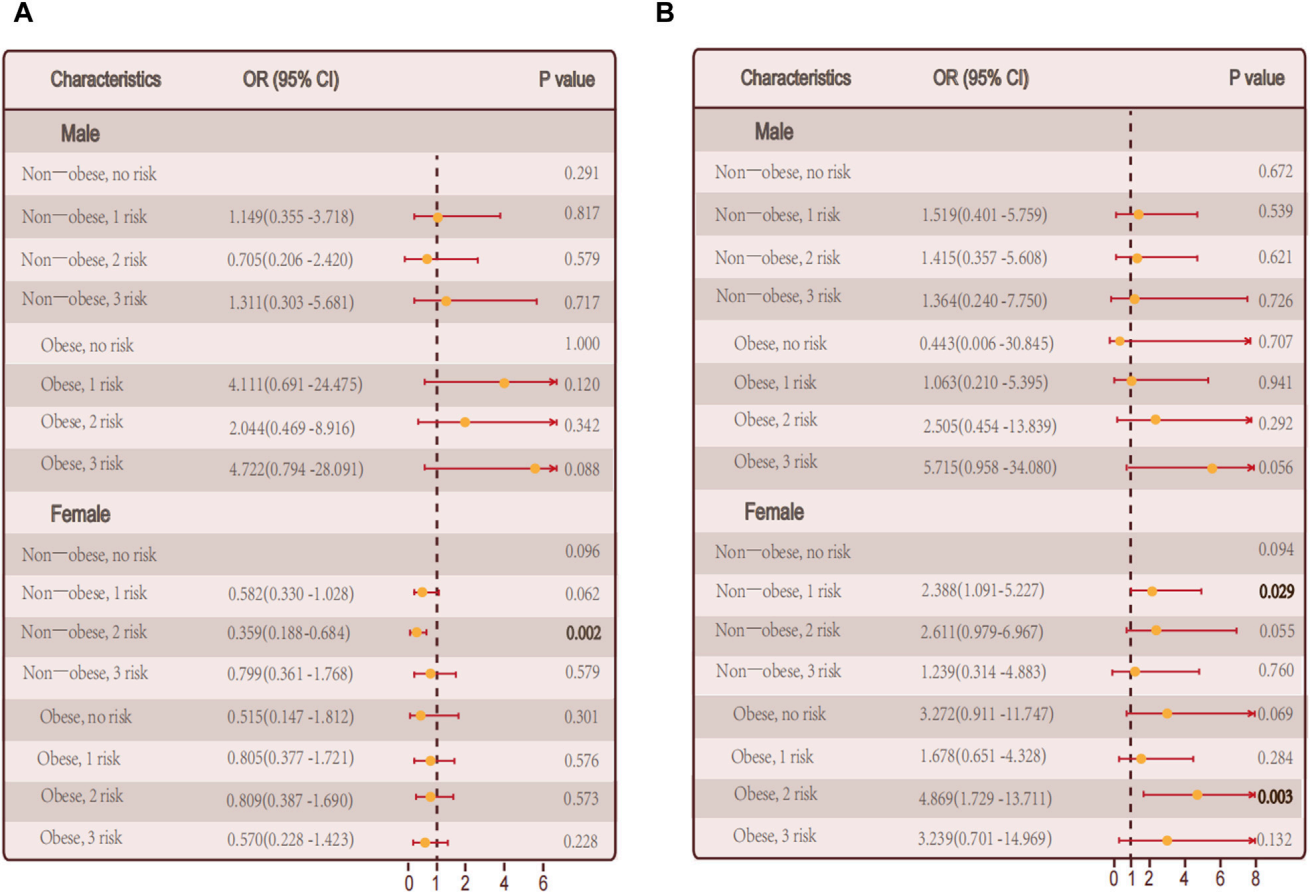
Relationship between hypothyroidism and the number of metabolic abnormalities in men and women. **(A)** The number of metabolic abnormalities and the number of hypothyroidism in men, women, and all individuals in the non-obese and obese population in persons with autoimmune thyroid disease. **(B)** In persons without autoimmune thyroid disease, Association of the number of metabolic abnormalities with hypothyroidism in all individuals in men, women, and non-obese and obese populations.

Then we performed differential analysis by gender. In male who did not have autoimmune thyroid disease, there was a notable correlation between the quantity of metabolic irregularities and hypothyroidism in female patients (*p* = 0.03) (Figure 4A). However, there was no notable correlation between the quantity of metabolic irregularities and hypothyroidism (Figure 4B).

Among autoimmune thyroid disease individuals, there was no notable correlation between the quantity of metabolic irregularities and hypothyroidism in male and female, respectively (Figures 4A, B).

#### 3.4.1 Logistic regression analysis in non-autoimmune group and autoimmune group

**FIGURE 5:**
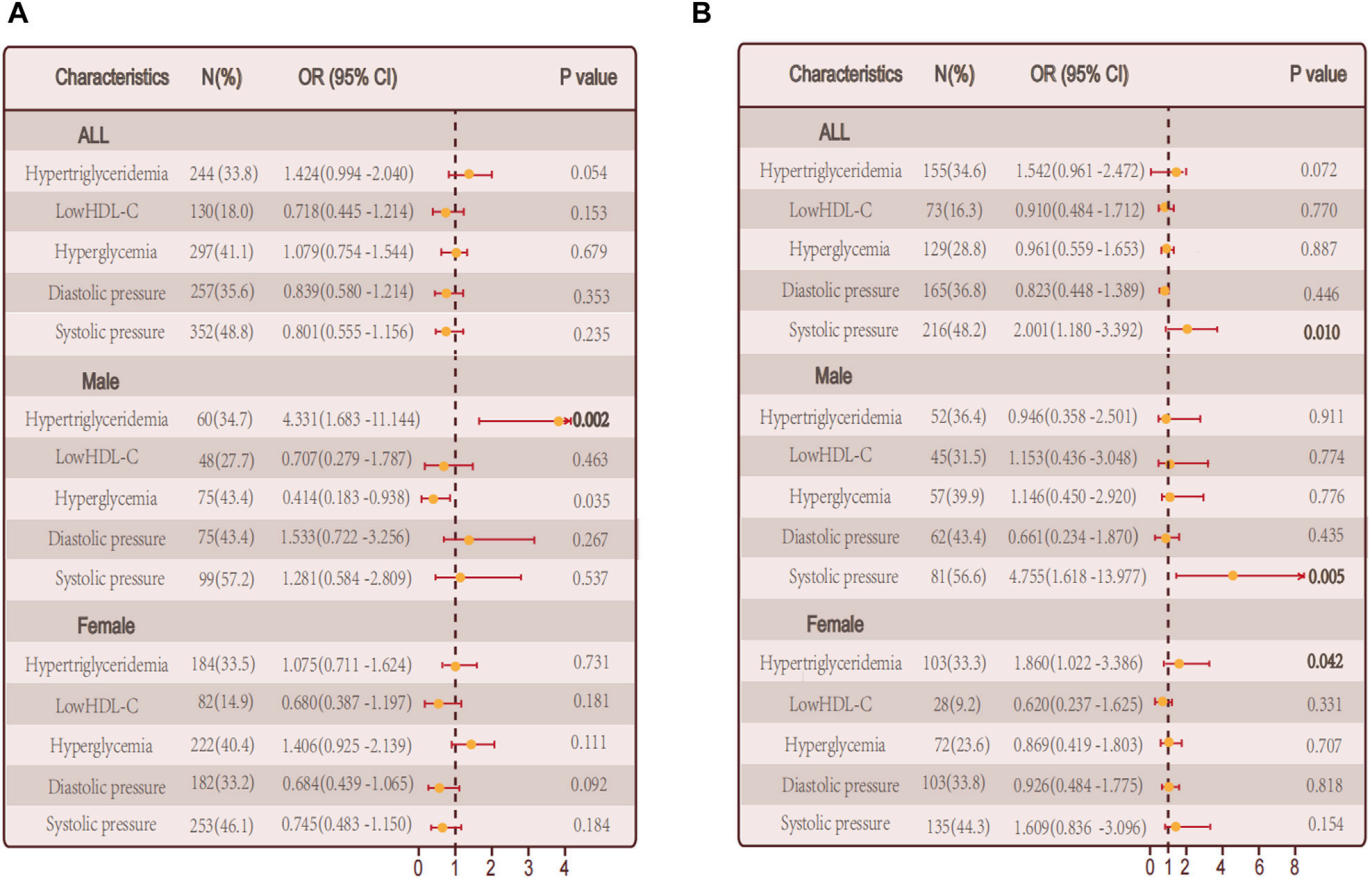
Relationship between single metabolic abnormalities and hypothyroidism in a population. **(A)** Relationship between single metabolic abnormalities and hypothyroidism in a population with autoimmune thyroid disease. **(B)** Relationship between single metabolic abnormalities and hypothyroidism in a population without autoimmune thyroid disease.

Combining this with our analysis of obesity metabolic phenotypes, we hypothesize that specific metabolic factors might be significantly related to hypothyroidism. Further exploration of individual metabolic factors and the risk of autoimmune thyroid disease and non autoimmune hypothyroidism through logistic regression analysis. In the group of individuals with autoimmune thyroid disease, there was no significant association between hypertriglyceridemia and hypothyroidism (*p* = 0.054). However, when considering gender separately, a significant positive correlation was found in males (*p* = 0.002). Additionally, fasting plasma glucose levels showed a significant correlation with hypothyroidism (*p* = 0.035) (Figure 5A). In the non-autoimmune group, systolic blood pressure was significantly correlated with hypothyroidism (*p* = 0.010), with similar findings in males (*p* = 0.005). Interestingly, in females of this group, hypertriglyceridemia was significantly correlated with hypothyroidism (*p* = 0.042) (Figure 5B).

## 4 Discussion

In this study, we investigated the relationship between different causes of hypothyroidism and metabolic obesity phenotypes using data from the Thyroid Disease Database of Shandong Provincial Hospital. Compared to MHNO phenotypes, our findings reveal that hypothyroidism and MUO are strongly and positively associated. This finding supports the previous research conducted by our team (Wang et al., 2021). Upon classifying the sample into subcategories of people with and without autoimmune thyroid disease, it was noted that within the non-autoimmune category, the MUO phenotype exhibited a noteworthy association with hypothyroidism, especially among females, whereas no such association was detected among males. On the other hand, among the autoimmune group, the occurrence of the MUNO phenotype was identified as a separate factor that increases the risk of hypothyroidism, particularly in females, while no notable association was found in males.

Previously, Cohort studies conducted by our team have examined how obesity metabolic types relate to hypothyroidism. We found that this relation remains unaffected by the presence of TPO-Ab positivity (Wang et al., 2021), aligning with earlier research (Cheng et al., 2022). In order to examine the correlation between 203 obesity metabolic characteristics and the occurrence of hypothyroidism, the current study further 204 classified the population into two cohorts: those with and without autoimmune thyroid disorders. A risk factor independent of thyroid function for hypothyroidism was discovered in the autoimmune MUNO group, notably in females.

Examining the association between the quantity of metabolic irregularities and hypothyroidism indicated that a rise in metabolic irregularities constituted a separate hazard element for hypothyroidism in the non-autoimmune category, whereas no such connection was observed in the autoimmune category. This suggests the potential impact of individual metabolic abnormalities on hypothyroidism. Additional examination revealed that increased levels of triglyceride (TG) were identified as a separate factor that contributes to the risk of hypothyroidism. Earlier research has shown a strong connection between underactive thyroid and high levels of fats in the blood, known as hyperlipidemia (O Brien et al., 1993; Amouzegar et al., 2020; Gao et al., 2015; Roos et al., 2007; Lai et al., 2011; Gutch et al., 2017). A positive correlation between TG levels and TSH levels is observed when TSH levels are normal. Due to thyroid hormone deficiency, enzymes involved in lipoprotein metabolism are less active, contributing to this correlation. Consequently, TG levels often experience frequent increases. Additionally, TSH has the ability to attach to the TSH receptor and stimulate the production of TG in specialized fat cells through the AMPK/PPARγ signaling pathway (Ma et al., 2015). Furthermore, hyperlipidemia could potentially trigger hypothyroidism through the suppression of thyroid hormone synthesis expression and/or function. In our previous investigation, it was discovered that palmitic acid has the ability to decrease the expression and functionality of three crucial molecules that play a role in the production of thyroid hormones (NIS, thyroglobulin, and thyroid peroxidase) in primary thyroid cells of humans. As a result, this has an impact on the functioning of the thyroid gland (Zhao et al., 2018). Moreover, endoplasmic reticulum stress may explain palmitic acid treatment’s inhibitory effects on these molecules (Wen et al., 2017; Zhang et al., 2019). Moreover, sterol regulatory element-binding proteins (SREBPs) have also been documented as innovative transcriptional regulators of Tg, NIS, and TPO in thyroid epithelial cells, besides controlling lipid synthesis and absorption (Ringseis et al., 2013; Rauer et al., 2014; Wen et al., 2016).

## 5 Conclusion

Our study found a significant correlation between obesity, metabolic problems, and abnormal thyroid function in patients without concurrent thyroid autoimmune disease. However, for thyroid autoimmune patients, it is crucial to pay attention to metabolic abnormalities, especially triglyceride levels. Therefore, further research is necessary to elucidate the process of thyroid autoimmune development and provide new perspectives for the effective treatment of hypothyroidism.

### 5.1 Limitations

There are specific constraints in this study that require attention. First of all, due to the fact that the study is cross-sectional, it does not adequately clarify the causal relationship between obesity metabolic phenotypes, metabolic factors, and hypothyroidism development. The causal relationship between metabolic obesity phenotype and hypothyroidism needs further follow-up. There is a need to investigate the causal relationship between metabolic obesity phenotype and hypothyroidism. Large-scale longitudinal cohort studies are needed to further elucidate causal relationships between metabolic obesity phenotype and hypothyroidism. Additionally, our definition of obesity is solely based on BMI, and the definition of metabolic obesity phenotype remains controversial. And since waist-hip circumference information was not obtained in this study, the effect of central obesity on hypothyroidism needs further attention. In conclusion, the relatively limited size of the sample requires the inclusion of extra external data to further validate and explore. The authors declare that the research was conducted in the absence of any commercial or financial relationships that could be construed as a potential conflict of interest.

## Data Availability

The raw data supporting the conclusion of this article will be made available by the authors, without undue reservation.
